# Formulation of new drug delivery systems for insulin from natural bioactive biocompatible polymers

**DOI:** 10.1038/s41598-025-86938-4

**Published:** 2025-01-31

**Authors:** Howida A. Fetouh, Engy E. A. Aleem, Najiyah H. Mohammed, Jihad M. Abd-Almajeed Aldesouky, Amel M. Ismail

**Affiliations:** 1 Chemistry Department, Faculty of Science, Alexandria University, Alexandria, Egypt; 2 Clinical Pathology Department, Faculty of Medicine, Alexandria University, Alexandria, Egypt

**Keywords:** Chitin, Guar gum, Insulin, Drug delivery system, Release, Rate, Biochemistry, Biotechnology, Drug discovery, Chemistry, Materials science

## Abstract

New insulin drug delivery systems (IDDs): insulin@chitin; insulin@chitin-grafted (g)-guar gum were prepared by using a modified sol–gel method. Insulin vials were loaded on the safe natural inert bioactive polymers (chitin and chitin-g-GG copolymer) carriers using water green solvent. Traces amount additives were below toxicity limits. Guar gum increased the numbers of the functional groups of the polymer carrier. Insulin release monitored at 37 ± 0.5 °C and buffer solutions of pH (1.2, 6.8 and 7.4) simulating physiological body fluids: stomach, intestine colon and blood stream. Insulin released from insulin@chitin only at pH 7.4. No release observed at pH 1.2, 6.8 due strong bonding to acetyl group of chitin. Insulin@chitin-GG system showed sustained targeting insulin-release at pH: 6.8 > 7.4 > 1.2. Release data obeyed pseudo second order kinetic model indicating that IDDs is heterogeneous solid surface of energetically different active sites. Each insulin molecule occupied two active sites. The slow release at pH 1.2 indicated protection against stomach juice. Release kinetic depend on physicochemical characteristics (porosity, swelling ratio as well as peptide and amino acid sequence). Both IDDs showed negative zeta potential indicating stability against aggregation. Gaur gum improved particle size distribution and insulin release.

## Introduction

Chitin is a natural film forming bioactive polymer, available, biocompatible and biodegradable^1^. Chitin is hypolipidemic, antimicrobial, wound healing and immunoadjuvant^2^. It is abundant in crustaceans shells of seaweeds, anthoropods exoskeleton such as lobster, shrimp, insects and cephalopods such as squid and octopus fish. Chitin was named as β-(1 → 4 glycoside)-N-acetyl-D-glucosamine), or 2-acetamido-2-deoxy-D-glucopyranose. The activity of chitin exerted by the primary alcoholic (CH_2_OH) and acetamide (CH_3_CONH) groups. Chitin chains form intra molecular hydrogen bonding (H.B). Chitin extracted as: collected shrimps scraped from loose tissue, washed and dried, demineralized in 15 wt.% HCl at room temperature (RT) for 36 h, filtered, washed thoroughly with tap- and demineralized water and dried at 80 °C, grounded and sieved to 80 meshes size. Twenty gram powder was added to 5% NaOH (deproteinization), agitated for 1 h at 90 °C, filtered, washed with tap water till neutrality, dried at 60 °C for 4 h.

Guar gum is a plant resin linear poly-β-1, 4 anhydro mannose-galactose residues contains 4 OH per monomeric unit^3^; widely used in biosorbents not in DDS^4^. Human insulin is a transporter dipeptide protein hormone of the molecular formula: C_257_H_383_N_65_O_77_S_6_. Insulin synthesized, releases and stored in the pancreas. The amino acids (aa) chains: A (21aa) and B (30 aa) residues linked by 2 disulfide bonds between cysteines units. Peptide bond formation via condensation gives the aa sequence. Blood insulin controls and regulates cellular or organs response to blood glucose level (BGL)^5^. After a meal, insulin releases into blood activates cellular absorption of blood glucose (BG) for growth and energy. Insulin binding cell membranes receptors, Glucose transporter proteins released from cell interior to cell membrane carry and transport BG into the tissue.

The IDDs tablets keeping are required for keeping of human health^6^. Insulin vials such as Lantus Glargine and Levemir Detemir are long acting human insulin. Glargine is 10aa to 50 aa protein regulates uptake BGL via binding, transport into or out the cell^7,8^. Diabetes Miletus (DM) chronic epidemic metabolic disease causes micro vascular complications such as retino-, nephron-, neuropathy and foot disorders. Subcutaneous insulin injection for types I, II Diabetes Miletus (T1DM and T2DM) showed many drawbacks such as discomfort, pain and local infections^8^. Acceptable insulin tablets mimic pharmacokinetics of endogenous insulin. Reported insulin carriers are pH-sensitive biodegradable biocompatible micro-particles and liposomes; layer-by-layer coat, polymer shells and nanocomposites (NCs). The obstacles of IDDs are the broad size distribution, unreproducible and uncontrolled initial burst release, excessive hypoglycemia and immunological response^9^. Examples of obstacles included: The microspheres poly (acrylic acid) @ chitosan-5-Fluoro-uracil required sodium-Na-taurocholate absorber^10,11^. Insulin leaking from the alginate-Ca(II) ions microspheres due to the broad particle size distribution^12^. Βeta cyclodextrin emulsions caused hypoglycemia^13^. The poly-(anhydride, p-(COOH-ethyl-formamido)-benzoic anhydride] showed toxicity^14^. Also the inefficient micro emulsion Eudragits adhered to the gastro intestinal tract (GIT)^15^.

Anionic poly methyl acrylamide CH_3_-ester release insulin only at high pH^16^. Burst release showed by encapsulated microspheres –OH-propyl-CH_3_-cellulose acetate succinate. Eudragit-poly-lactic acid microspheres showed low entrapment efficiency and enzymatic degradation. Microspheres: good absorption: Tri-CH_3_-chitosan-Eudragit- Mg-stearate required lubricant^17^; OH-propyl-CH_3_-cellulose phthalate-Na-*N*-(8-[2-OH-benzoyl]-NH_2_ should contain absorption enhancer^18^; Macromolecule poly ester amide limited by the enzymatic hydrolysis^19^.

Chitosan (CT) increased insulin contact with duodenum and jejunum but low absorption^20^. It is a safe biocompatible biodegradable polysaccharide drug carrier, however the positive charges on its amino (NH_2_) group electrostatically attracted to the negative charges on insulin forming insoluble complex. In addition, CT has basic characteristics (pKa ≈ 6.5), so it dissolves in the acidic media. Hence CT-insulin insoluble complex tend to be soluble in the acidic juice of gastro intestinal tract^6^. CT required grafting by another polymers to be insoluble. The plan of using chitin as a new carrier for insulin was based on the bond formation of insulin with the acetyl group of chitin. Chitin could be new more suitable polymer carrier for insulin than chitosan. This study aims formulation and evaluation of a new oral administration IDDs using natural inert biocompatible polymer chitin and GG carriers to overcome all the reported obstacles.

## Experimental

### Materials

Analytical grades pure chemicals purchased from certified companies of chemical products used without further purifications. Chitin A.R. (Flakes (C_8_H_13_NO_5_)_n_ average molecular weight ($\overline{{M_{w.} }}$ 17–20 kDa, (monomer Mw.259 Da), Batch NO. CH 546: Alpha Chemika Co. Powder GG (Mw. 10 k Da): Loba Chemie Co.-Mumbai-India. HCl, NaOH and glutaraldehyde crosslinker: EL Gomhoria Co. Ammonium persulphate (NH_4_S_2_O_8_) and sodium dodecyl sulphate (SDS) anionic surfactant catalyst and insulin Humalog vial solution lispro (3 mL (3.5 mg, 100 units’ mL^−1^: Sigma Aldrich Co. The chemical structure of insulin carrier was represented supplementary information (SI), Fig.SI.1.

### The mechanism of formulation insulin drug delivery systems

The insulin drug delivery systems (IDDs) were composed of compatible constituents. The type and the amount of the constituents of IDDs were shown in Table 1.

**Table 1:** Chemical constituents IDDS.

Physical properties			Insulin	Chitin	GG	Glutaraldehyde
Insulin	Yellow color	%Yield	Wt.(g)			
@chitin	Pale	86.1	3 mL	2	–	0.1
@chitin-GG	Dark	75.2		1	1	

Traces amount of NH_4_S_2_O_8_ (0.05g) as free radical initiator and 0.03g SDS emulsifying agent below the toxicity limits were added. The IDDs were prepared in three neck Pyrex glass cell of volume capacity 100 mL following the sol–gel method. The constituents’ materials were mixed and dissolved in 40 mL double distilled water. Alkaline pH 8 adjusted by 0.1 M NaOH and 0.1 M HCl. The reaction mixture was magnetically stirred at 150 rpm, 40 °C for 3.0 h under inert nitrogen (flow rate 10 mL min^−1^). Then the mixture was cooled to the room temperature. Then the mixture set to equilibrium overnight. The polymer chitin and guar gum suspend in water by the trace amount of the SDS surfactant. The contact time between insulin vial and the carrier is sufficient for efficient loading. Insulin incorporated on the carrier chitin or chitin-guar gum via integration of insulin with nanoparticle of chitin and guar gum during the formulation time. The reaction mixture was filtered under suction. The solvent was evaporated at 55 °C under reduced pressure. The nanoparticles of IDDS collected through centrifugation at 14,000 rpm for 20 min. to remove the excess solvent. The free insulin and surfactant washed away with double distilled water to remove the remaining unreacted constituents. The obtained solid products were grinded into a nanoscale powder using mortar and pestle and packed in the sealed vials^12^.

High insulin drug loading in the formulation drug delivery systems could reduce the amount of matrix drug carrier required. The loading mechanism involved adsorption or desorption processes of insulin into the pores of the polymer carriers. The drug loading and encapsulation efficiency were ensured due to good solubility of insulin in the carrier and excipient matrix that depend on the matrix composition, molecular weight, insulin-polymer interaction and the presence of functional groups (hydroxyl and acetyl group) in the drug matrix. The presence of guar gum in the carrier matrix improved the ionic interaction between insulin and matrix materials by the addition of further hydroxyl functional groups.

### Characterization of the drug delivery systems

The functional groups (FGs) were confirmed using Fourier Transformer Infrared (FTIR) spectroscopy (KBr) using Bruker TENSOR 37 spectrophotometer, Model 1430 PS calibrated (1602 ± 1 cm^−1^) at the frequency range 4000–450 cm^−1^ at RT^6^. Scanning electron microscope (SEM) micrographs using JSM-IT200 SEM. Powder X-ray diffraction (PXRD) patterns at 25 °C using Cu-Kα radiation of wavelength (λ) 1.54060 Å accelerated at 40 kV using Bruker D8 advance diffractometer: Angle range& rate were 5–70°, 0.02o step, 1° min^−1^ respectively^6^. Differential thermal- and thermogravimetric analysis (DTA), (TGA) respectively using Shimadzu DTA/TGA-50, heating rate 10 °C min^−1^ using Pt. cell under nitrogen rate flow 20 mL min^−1^. TGA plot (weight loss sample as a function of (temperature or time) and DTA (weight loss versus temperature difference. ($\Delta {\text{T = T}}_{{{\text{sample}}}} - {\text{T}}_{{{\text{inertrefrence}}}}$) at zero difference heat flow against heating temperature (t °C)^6^. Mean swelling ratio ± SD, n = 3 in deionized water at RT due to water absorption into pores was determined using Eq. 1 as: lyophilized sample was swelled in excess phosphate buffer saline (BFS), pH 7.4, 37 ± 0.5 °C, time intervals (5–60 min. and 1, 8, 15 days; removed from BFS, reweighed after removing the excess surface H_2_O using blotting paper. The swelling ratio was calculated using Eq. 1^21^:

1 $${\text{Swelling}}\;{\text{ ratio }} = \frac{{W_{s} - W_{o} }}{{W_{o} }} \times 100$$

where ${W}_{o}$, ${W}_{s}$ are the weight of the dry and the swollen sample respectively.

Mean porosity percent ± SD of lyophilized sample was determined in triplicates (using Eq. 2) at 37 ± 0.5 °C by liquid displacement method; 0.2 g sample was immersed in 10 mL distilled water $( \text{density }\rho )$ 24 h till equilibrium; freeze dried and reweighed^22^:

2 $${\text{Porosity }}\% \, = \frac{{W_{2} - W_{1} }}{{\rho V_{s} }} \times 100$$

where ${W}_{2}$, ${W}_{1}$ sample weights before and after immersion, ${V}_{s}$ volume of dry sample.

Insulin denaturation was done by 2 mL Biuret reagent (0.39 g hydrated CuSO_4_ + 9 g Na–K- tartarate) in 500 mL 0.2N NaOH to sample, vortex mixing, RT incubation 15 min. Absorbance at 550 nm was measured^23^. The amino acid (aa) contents were determined as follows: Different volumes standard aa glycine was pipetted into labeled test tubes. 1.0 mL ninhydrin reagent was added to all sample (blank and unknown), contents are mixed by vortex, placed in boiling water in waterbath for 15 min. 1 mL ninhydrin was added, mixed well and the absorbance was recorded at 570 nm. Calibration curves of Burette and Ninhydrin tests were used^24^. Particle size (PS) of diluted sample suspension with distilled water to appropriate concentration was determined in triplicates using photon correlation spectroscopy (PCS), NanoZS/ZEN3600 Zetasizer via non-invasive backscattering technology at 173° detection angle at 25.0 ± 0.1 °C. Zeta potential was determined of diluted sample. Volume of 1.0 mL suspension is placed in a universal folded capillary cell contains Pt. electrodes^25^.

### Kinetic of invitro insulin release

The standard calibration curve of Insulin was constructed using known concentrations (7, 10.5, 14, 17.5, 21, 24.5, 28 and 35 ppm) diluted from 10 mg/mL insulin in 100 mL absolute ethanol^26^. Insulin release from IDDSs was monitored by recording UV–vis. absorbance (A) at 274 nm, double beam spectrophotometer UV T80. Constant length dialysis bag: (VISKING dialysis cellulose tubing 21 mm diameter was soaked 10 min. in distilled water at RT, rinsed thoroughly with distilled water to remove preservative^27^; 30 mg sample was dissolved in 1 mL buffer solutions at 37 ± 0.5 °C; placed in 25 mL buffer solution in waterbath agitated at100 rpm. Released insulin was determined via withdrawing 3.0 mL releasing medium at different times. Total buffer volume was kept 25 mL. Release from insulin @chitin cannot be monitored at pH 1.2, 6.8 due strong linkage of peptide amide bond on insulin to acetyl group of chitin. Release data in duplicates from insulin@chitin-g-GG were linearly fitted to kinetic models: Zero order (Eq. 3): slow release from non-disaggregate DDs^16^.

3 $$Q_{t} = Q_{o + } K_{o} t$$

Q_0_ (= 0), Q_t_ are the initial and released concentration respectively, K_0_ rate constant oncentration time^−1^).

Pseudo 1° (Eq. 4): Concentration change with time^16^.

4 $${\text{ln}}(q_{e} - q_{t } ) = {\text{ln}}(q_{e} ) - k_{1 } t$$

where *q*_*e*_, q_t_: equilibrium and time dependent desorption capacity respectively (mg g^−1^), k_1_ rate constant (min^−1^).

Pseudo (2°), Eq. 5^16^:

5 $$\frac{t}{{q_{t} }} = \frac{1}{{k_{2 } q_{e}^{2} }} + \frac{t}{{q_{e} }}$$

where q_e_ and q_t_ equilibrium and time dependent desorption capacity (pp mg^−1^), respectively, k_2_ rate constant (g/mg min).

Initial desorption rate, h (ppm g^−1^ min^−1^) = *k*_*2*_*q*_*e*_^*2*^*.*

Elovich model: slow desorption chemisorbed insulin from heterogeneous surface, Eq. 6^28^

6 $$q_{t} = \frac{1}{\beta }\ln \left( {\alpha \beta } \right) + \frac{1}{\beta }{\text{ln}}\left( t \right)$$

where *α*: initial desorption rate (mg g^−1^ min^−1^), *β:* desorption constant (g mg^−1^) at the release time (t).

Huguchi (Eq. 7): Release from porous planar polymer; initial concentration is larger than released concentration, 1D constant diffusion without edging; particles smaller than system thickness; negligible swelling and carrier dissolution in perfect release media^28^.

7 $$q_{t} = {\text{dissolution constan}} (K_{H} ) \times t^{\frac{1}{2}}$$

Finger-Puppets-Korsmeyer-Peppas model (Eq. 8) represented drug release from a modified structural and geometrical carrier^16^:

8 $$log\frac{{C_{t} }}{{C_{\infty } }} = \log k + n\log t$$

(C_t_/C_∞_ = fraction release at time t, k: rate constant, n: diffusion exponent reflect transport mechanism, k: constant (t^n^ unit).

Insulin release from solution 50 ppm IDDs in buffer solutions was monitored using electrochemical impedance using determined using electrochemical cell at 37 ± 1 °C^29^. Gamry Potentiostat-G300)-sequencer V6.12, DC105 at the frequency range 100 kHz-10 Hz. AC signal 10 mV at open circuit potential using carbon felt working electrode (WE: cross sectional area 0.28 cm^2^ (cleaned by 1:1 V/V mixture 1.0 M HCl-deionized water, 1:1 (V/V) ethanol–water solvent 5 min., sonicated (2 min. at 47 kHz) in DI water, air dried), activated by agitation in 1.0 M H_2_SO_4_ for 24 h. Sulphate functional groups were adsorption site for the released insulin). Pt. wire and Ag/AgCl_(s)_ were used as counter and reference) electrodes respectively.

## Results and discussion

### Characterization of insulin drug delivery systems

**Fig. 1:**
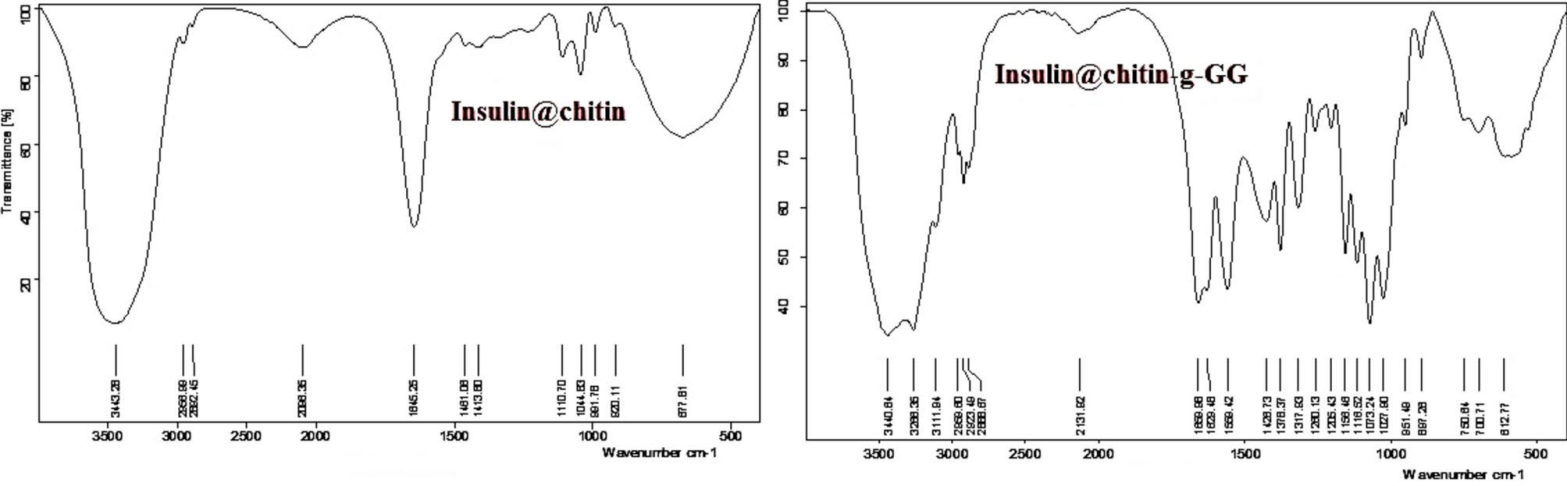
FTIR spectra of IDDS.

Figure 1 showed the different IR spectra confirmed modified chemical structure of chitin by grafted GG. Insulin@chitin-GG showed FGs chitin: CH_2_OH, CH_3_CONH), insulin: NH_2_, COOH. Assigned vibrational bands (Table SI.1) at characteristic wavenumber, ($\overline{\upsilon },$ cm^−1^) confirmed intercalation between chitin and GG^30^.

The vibrational bands around 3444.6 due to presence of –OH stretching and amine N–H symmetric vibrations. The band at 1070.4 cm^−1^ is due to –C–O groups stretching vibrations of N-acetyl group of chitin (the main adsorption center for insulin).The peaks between 1070.4–1028 cm^−1^ and 530–540 cm^−1^ indicated the presence of polysaccharide rings with the characteristic C–O groups. For insulin, O–H stretching, –COO– symmetric and asymmetric stretching, and –C–O–C-stretching appeared at 3239 cm^−1^, 1405 cm^−1^, 1592 cm^−1^, and 1024 cm^−1^ respectively. The C–O stretching vibration and NH bending with contribution of CN stretching vibrations at 1639 cm^−1^ and 1542 cm^−1^ respectively. GG exhibited the characteristic absorption bands at 3383 cm^−1^ and 2925 cm^−1^ due to O–H stretching vibrations associated with C-H stretching vibrations. In spectrum of insulin nanocomposites, two characteristic absorption peaks of insulin are almost masked and a new peak at 1594 cm^−1^ of C–N, mainly due to C–O stretching vibrations were observed^21^. The C–O stretching vibration and N–H bending with contribution of C–N stretching vibrations at 1639 cm^−1^ and 1542 cm^−1^ respectively^21^. In insulin@chitin-g-GG, two characteristic absorption peaks of insulin are almost masked, and a new peak at 1594 cm^−1^ of C–N, mainly due to C–O stretching vibrations are observed. Acloholic CH_2_OH group of chitin is also involved in H.B.with GG. Successful insulin loading was accomplished in according to FTIR spectra. Chitin contains hydroxyl (OH^**−**^) group of functional (CH_2_OH) groups at C2 of glucose monomer unit OH^**-**^ linked to carbony group of insulin-COOH on alkaline hydrolysis.

**Fig. 2:**
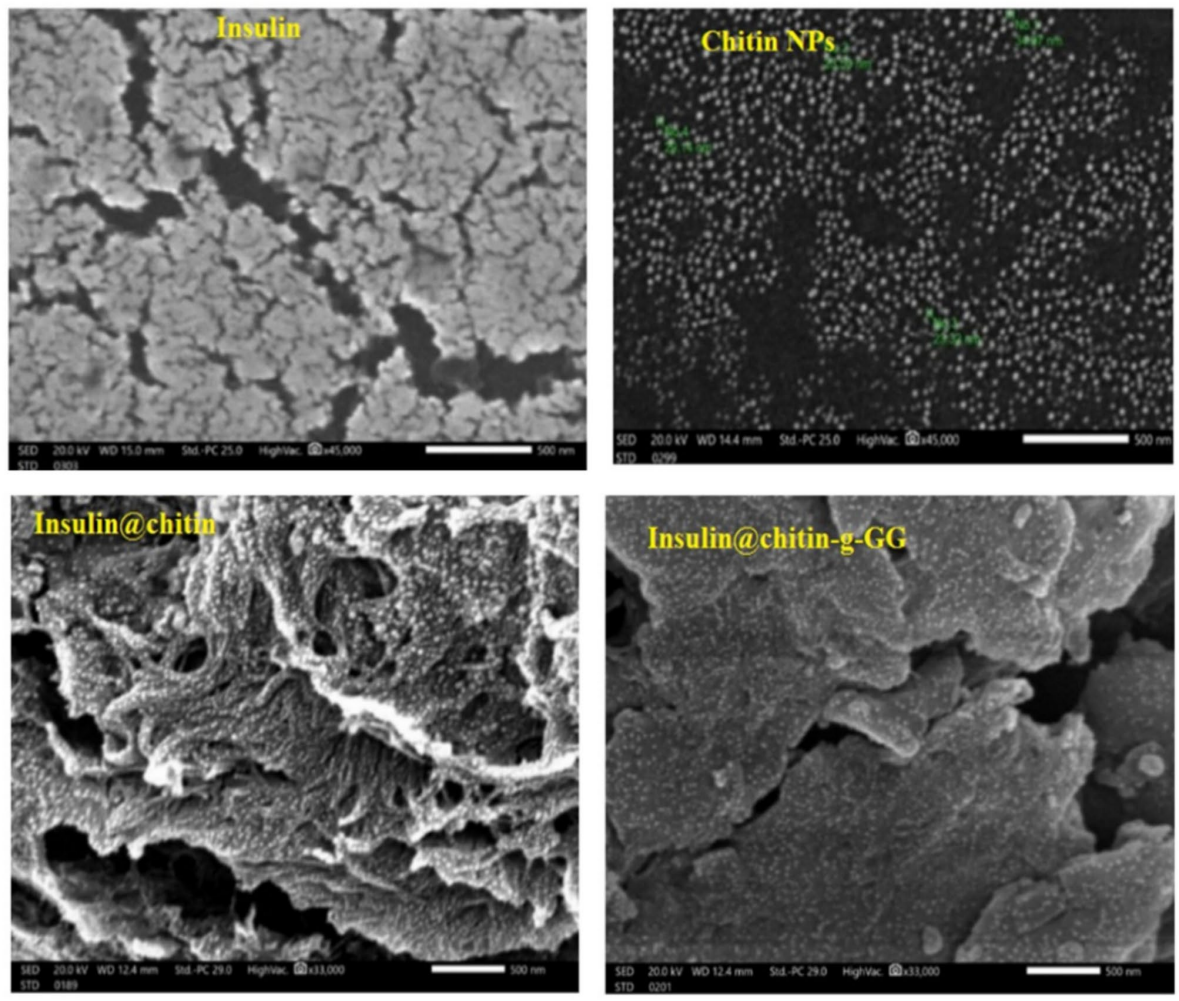
SEM micrographs, magnification 45,000 × .

Figure 2 showed SEM micrographs of insulin formulations in comparison to that of insulin and chitin.

Layered microstructure insulin due to interconnected polypeptide chains by: S–S bond, H.B between amino (NH_2_)–COOH of cysteine^6^. Bright fine spherical chitin nanoparticles (NPs) regularly linked insulin chains. Chitin and insulin morpholgy were maintained in IDDS. Copolymer is smoother carrier. Insulin added uniformly distributed on the carrier polymer with no coagulation.

**Fig. 3:**
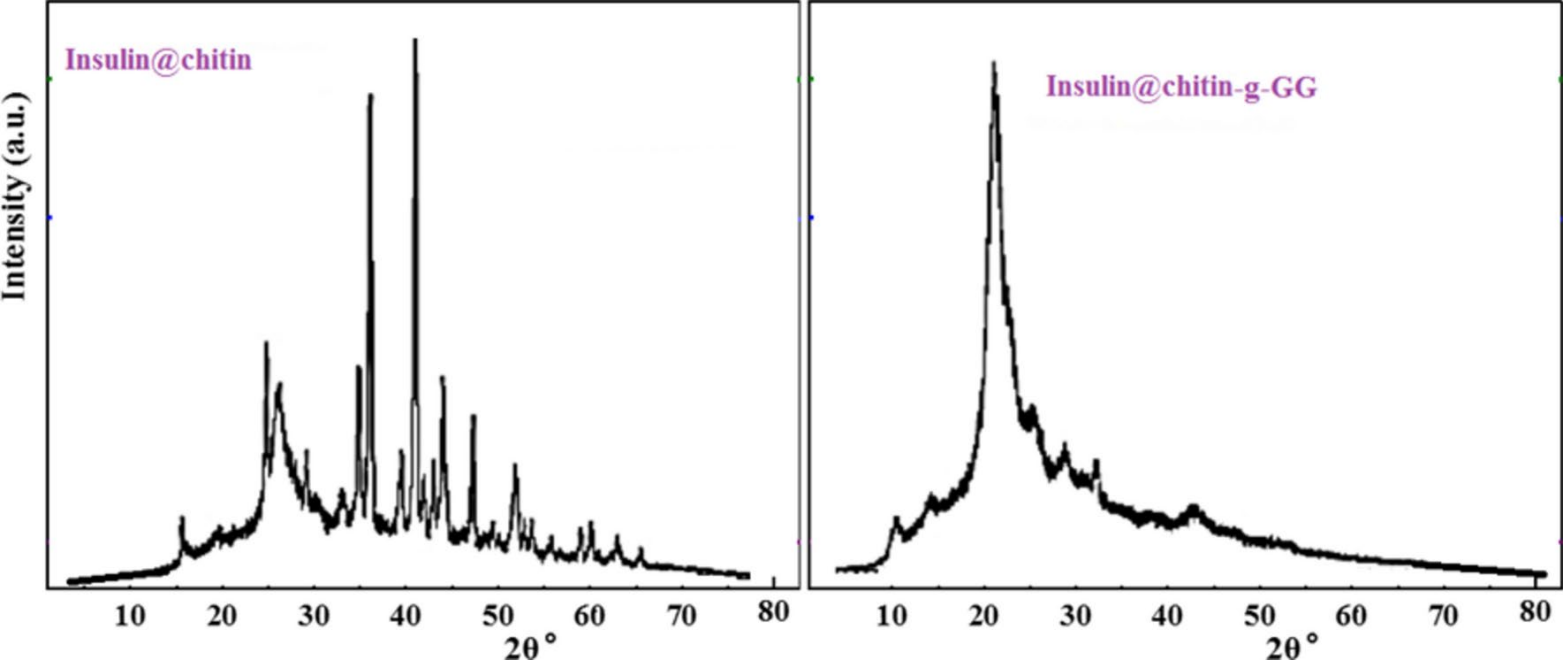
pXRD patterns of IDDs.

The pXRD patterns of the formulated IDDs were shown in Fig. 3.

The pXRD pattern of insulin@chitin showed semi-crystallinity characterizing native chitin Insulin@chitin is semi-crystalline (N atom of chitin is strongly bidentated O atom of COOH function group of insulin. Insulin@chitin-g-GG has both crystalline and amorphous domains^31^. Grafting chitin by GG declined chitin crystallinity. Higher crystalline insulin@chitin indicated that N atom of (NH) group of chitin is strongly binding O atom of COOH insulin in bidentate chelating. Binding GG to both insulin and chitin involved H.B. and Van der Waals interaction. Many diffraction patterns were observed in pXRD of insulin@chitin indicating more crystallinity than in insulin@chitin-g-GG. The only one broad diffraction peak was due to packing of GG into chitin carrier.

**Fig. 4:**
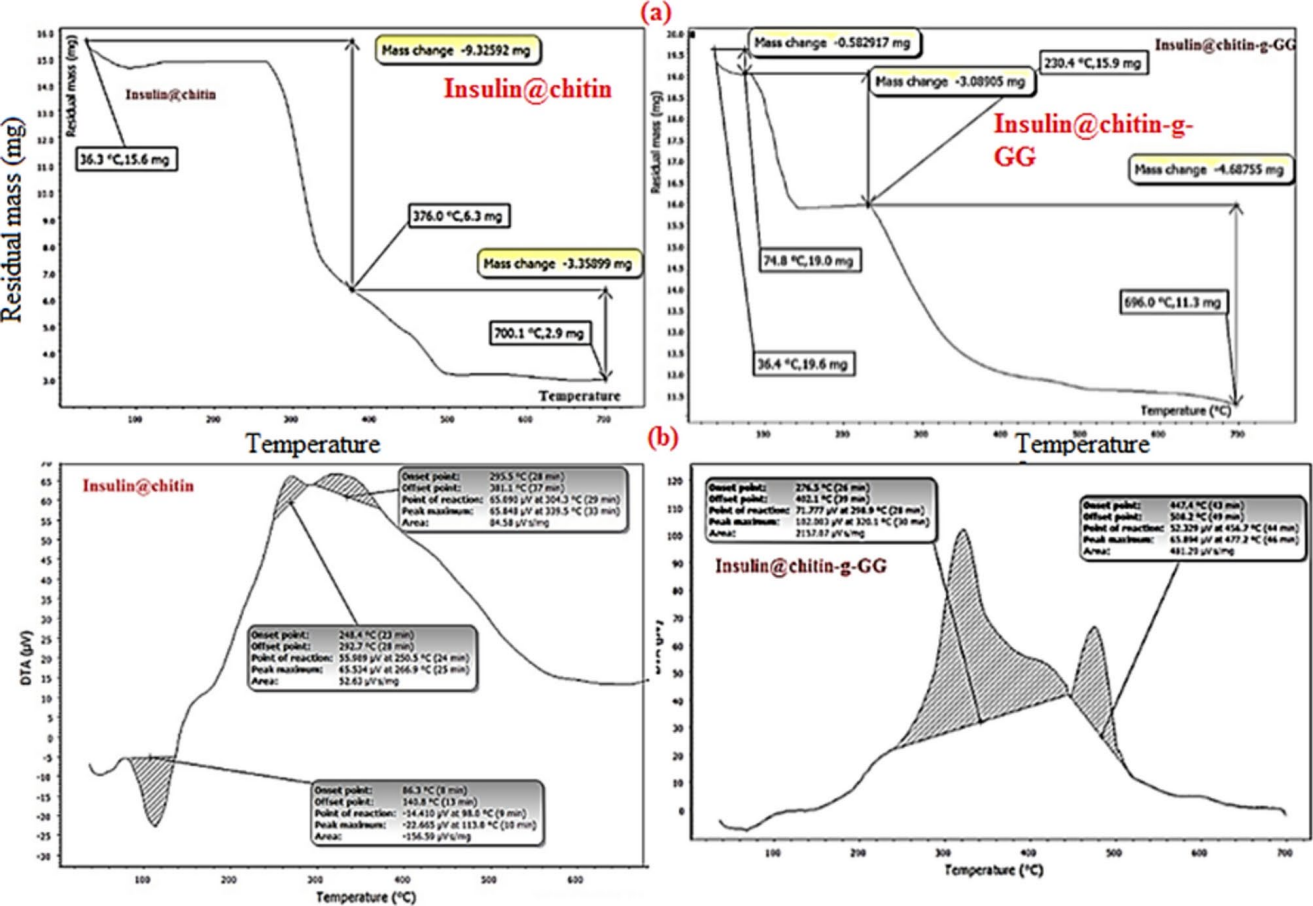
Thermograms of IDDs.

Figure 4 showed TGA and DTA thermograms. Insulin@chitin was better thermally stable than Insulin@chitin-GG. Thermal decomposition parameters were collected in Table 2^32^.

**Table 2:** Thermal decomposition data.

Insulin	Steps	t, °C range	Wt. loss%	%Residue	DTA, peaks t, °C
@chitin	2 2 3	36.4–74.8 74.8–230.4 230.4–696.0	3.45 18.30 27.74	66.86	86.3–140.8 (endo, intense) 248.4–292.7 (exo weak) 295.5-381.1 (exo weak)
@chitin-GG	1 2	36.3–376.0 376.0–700.1	59.78 21.53	18.59	276.5–402.1 (exo, broad& intense), 447.4–508.2 ( exo, broad& intense)

Insulin@chitin showed multistep decomposition and one endothermic peak at 86.3–140.8 °C due to strong bound FGs of chitin and insulin. Insulin@chitin-g-GG decomposed in 2 steps, higher wt. loss% and lower residue. Two broad intense exothermic peaks due to bonded decomposed carboneous polymeric residues species^33^.

### The validation of UV–visible spectroscopy for in vitro kinetics of insulin release

For quantification of insulin by absorption spectroscopy, the full wavelength scanning spectrophotometrically.The absorption maxima was found at maximum wavelength (λ_max._ 274 nm).The measurement of insulin release from the drug delivery system was validated using UV spectroscopy. The following section showed the complete details about the release study.

The validity of UV–visible spectroscopic technique was examined by constructing calibration curves of standard solutions (7, 10.5, 14, 17.5, 21, 24.5, 28 and 35 ppm) of insulin and recording the absorption of the full curves at λ_max._ 274 nm) as illustrated in experimental section "Kinetic of invitro insulin release".

**Fig. 5:**
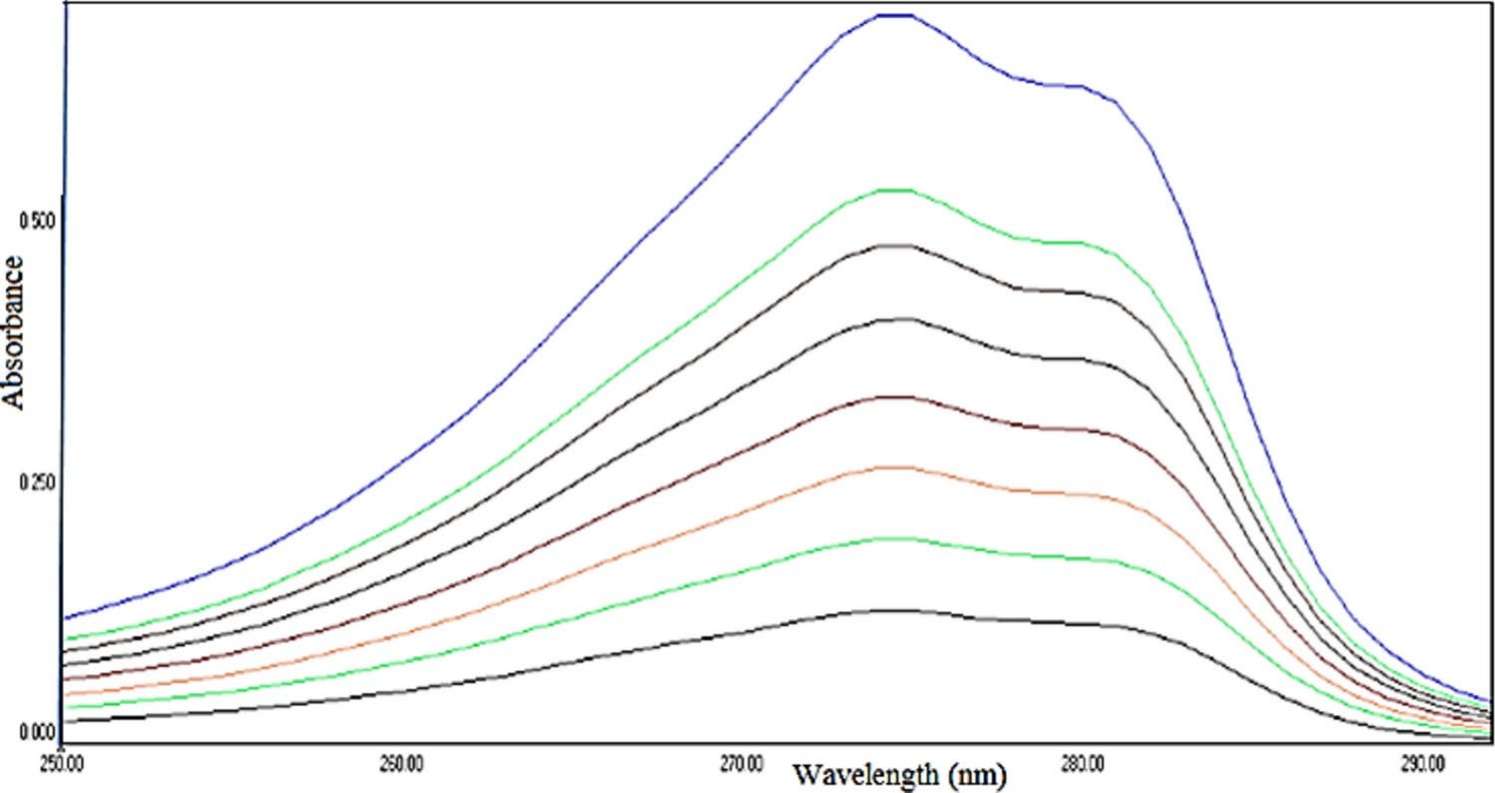
Full curves UV–vis. absorbance spectra.

Figure 5 showed UV–vis. spectra of the standard insulin solutions used in the construction of the calibration curve.

**Fig. 6:**
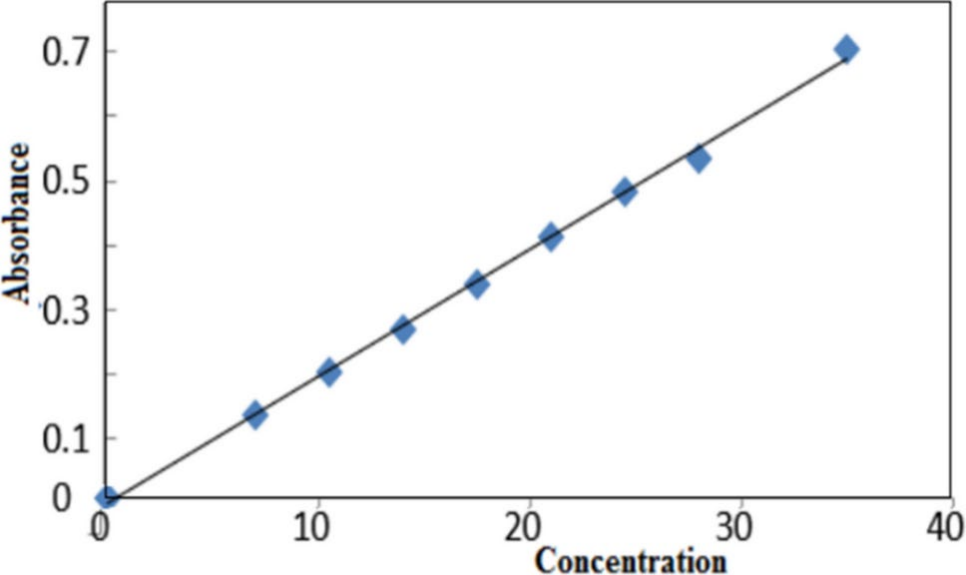
Standard calibration curve of insulin.

On applying the least square method on absorbance-concentration plot (standard calibration curve, Fig. 6), a good straight line was obtained with correlation coefficient equal 0.9984 confirmed the validity of UV–visible spectroscopy as an analytical method for analysis of insulin release.

**Fig. 7:**
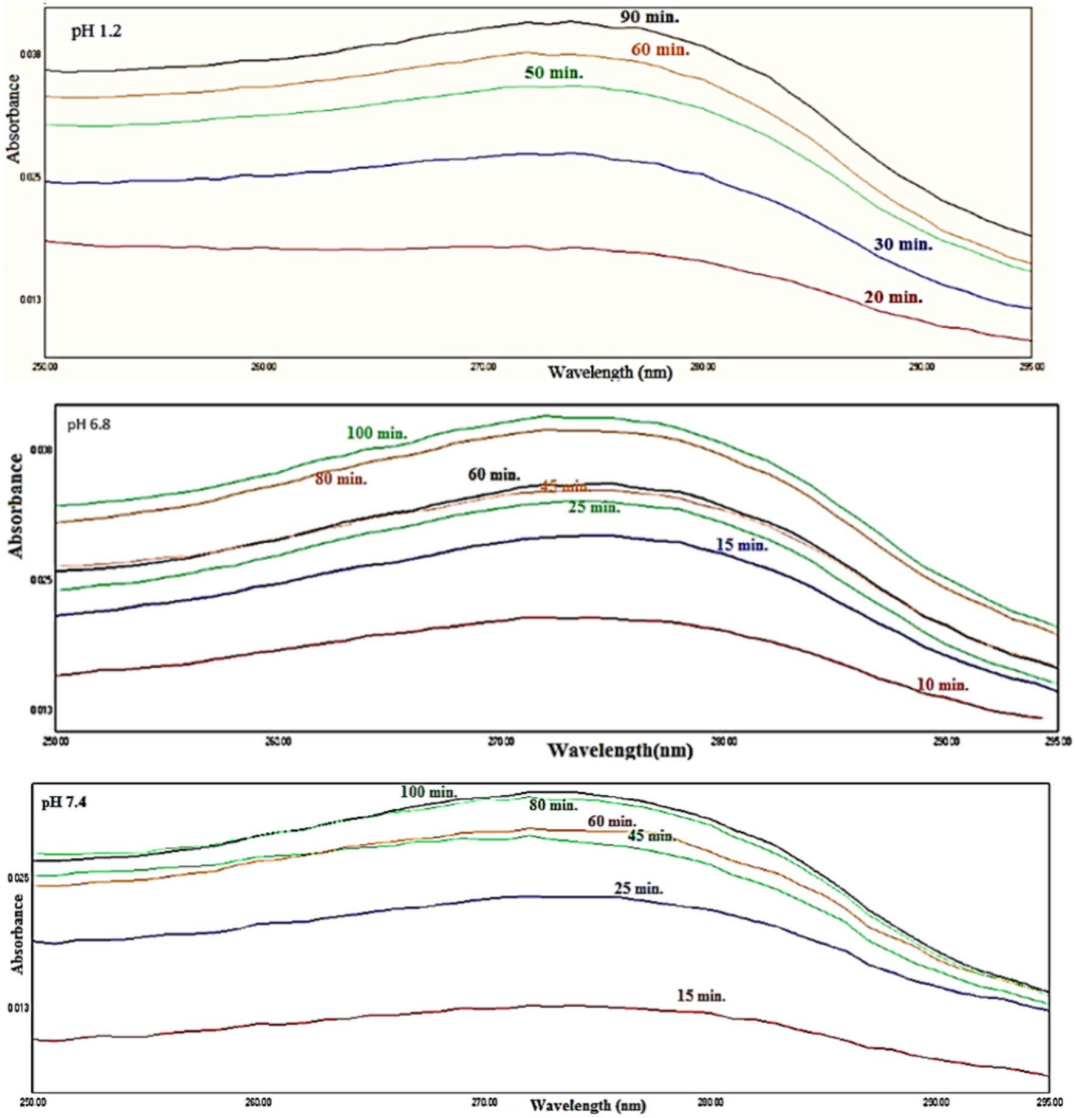
Variation of absorbance with time of insulin release at different pH.

The molar extension coefficient (ε) equals was calculated 0.005 from the slope of the obtained straight line. Figure 7 showed UV absorbance spectra of insulin released in the supernatant solution at λ_max._ 274 nm for insulin release from the nanocomposite insulin-chitin-g-GG at the different aqueous buffer solutions of pH (1.2, 6.8, and 7.4 simulating the biological fluids in the stomach, colon, and blood respectively at the physiological temperature 37 ± 0.5°C.

The insulin release from different DDS samples was assayed at λ_max._ 274 nm. Each experiment was performed in duplicates. Insulin release from insulin @chitin microcapsule cannot be monitored spectrophotometrically except at pH 7.4 due the strong linkage of peptide amide bond of insulin to the acetyl group of chitin. The concentration of insulin released was obtained using Beers Lambert law^26^.

**Fig. 8:**
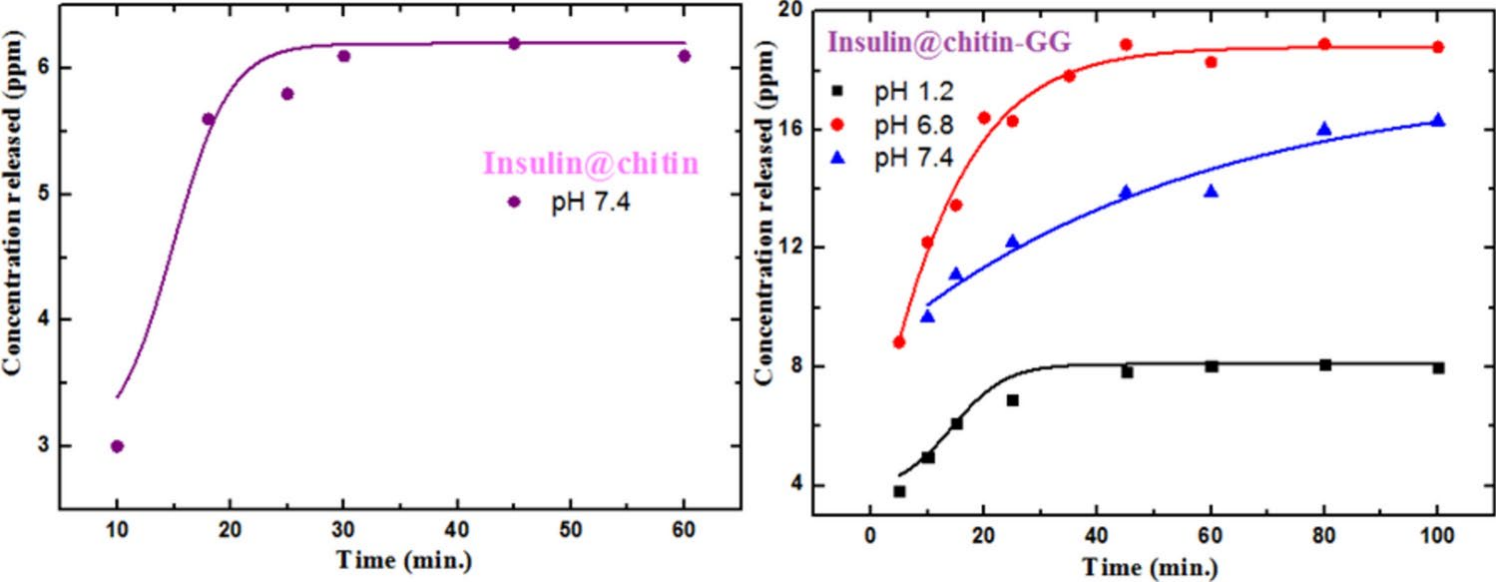
Released insulin concentration with the time.

Figure 8 represented the amount of insulin release from the two formulated drug delivery systems.

**Fig. 9:**
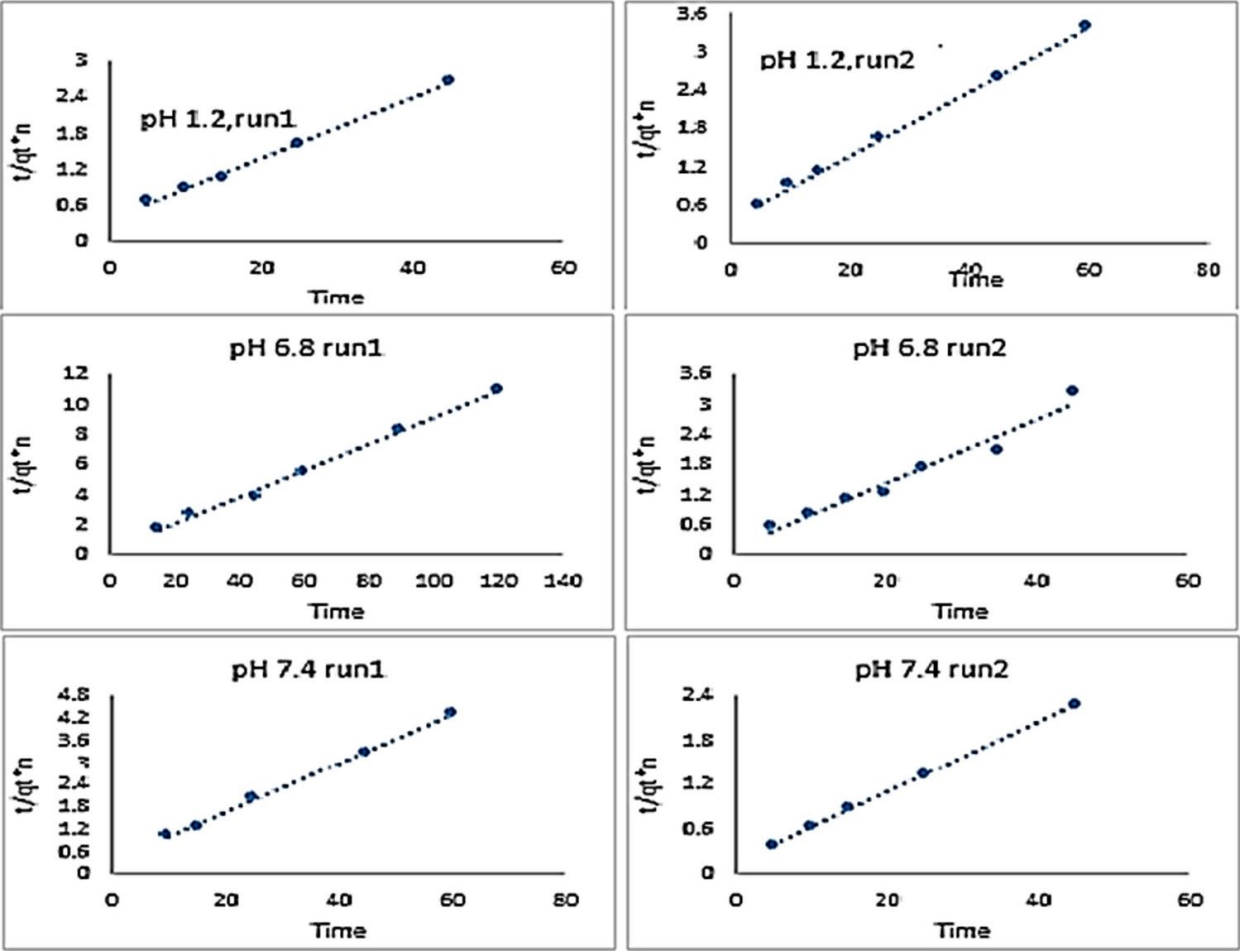
The linear fitting of the release insulin concentration to pseudo 2° kinetic model.

Insulin@chitin showed only insulin released at pH 7.4 give low 5 ppm concentration. Release at pH 1.2 and 6.8 cannot be followed spectrophotometrically. Insulin@chitin-g-GG showed higher release 10, 16, 18 ppm at pH 1.2, 7.4, 6.8. Encapsulated adhered insulin to permeable membrane polymer shell rapidly burst release (in 15 min.) detached in response to pH^21^ in agreement with half life time (few min.) pharmacokinetic of insulin in blood^27^. Insulin@chitin-GG showed controlled release limited at 76.2%, 80.7% and 67.2% at pH 1.2, 6.8 and 7.4 indicating stability against proteolysis. The release data from insulin@chitin-g-GG were linearly fitted to different kinetic model as represented in Fig. 9.

The least square linear regression analysis was applied. Table 3 collected correlation coefficients values (R^2^).

**Table 3:** Values R^2^ for linear fitting release data to kinetic models.

pH	Run	0°	Pseudo 1°	Pseudo 2°	Elovich	Higuchi	Ritger-Peppas
1.2	1	0.7259	0.8845	0.9965	0.943	0.8452	0.897
	2	0.8338	0.974	0.9994	0.9857	0.9261	0.9609
6.8	1	0.3691	0.3611	0.9959	0.5906	0.4794	0.6197
	2	0.3977	0.3821	0.9618	0.6574	0.5293	0.6915
7.4	1	0.7466	0.7549	0.9975	0.8645	0.8098	0.8368
	2	0.7721	0.7889	0.9998	0.9652	0.8815	0.9432

Based on the value of the correlation coefficient (R^2^), it was found that the best kinetic model fitted the release data was the pseudo second order model. Table 4 collected the results of the linear regression analysis (R^2^) and the standard error (S.E) for runs 1, 2 respectively.

**Table 4:** Parameters of regression analysis of released insulin to 2° model.

pH 1.2			pH 6.8			pH 7.4		
Regression eq.	R^2^	S.E.	Regression eq.	R^2^	S.E	Regression eq.	R^2^	S.E
y = 0.051x + 0.36	1.0	0.22	y = 0.088x + 0.256	1.00	0.32	y = 0.066x + 0.325	1.00	0.46
y = .050x + 0.374	1.0	0.25	y = 0.063x + 0.138	0.96	0.22	y = 0.048x + 0.140	1.00	0.58

The pseudo 2° model had the highest R^2^: (0.9618–0.9998) for all runs. It is the suitable fitted kinetic model for the release data of insulin. Non fitted Higuchi plot indicated desorption mechanism from polymer matrix is not under diffusion control^16,29^. The kinetic parameters *k*_2_, *q*_e_ collected in Table 5.

**Table 5:** kinetic parameter of pseudo 2° model.

pH	Run	k_2_ (g/mg min)	q_e cal_ (ppm)	h (ppm g^− 1^ min^−1^)	*R*^2^
1.2	1	0.0073	19.64	2.816	0.9965
	2	0.0067	19.92	2.674	0.9994
6.8	1	0.0305	11.32	3.908	0.9959
	2	0.0290	15.79	7.237	0.9618
7.4	1	0.0134	15.12	3.063	0.9975
	2	0.0161	21.05	7.133	0.9998

**Fig. 10:**
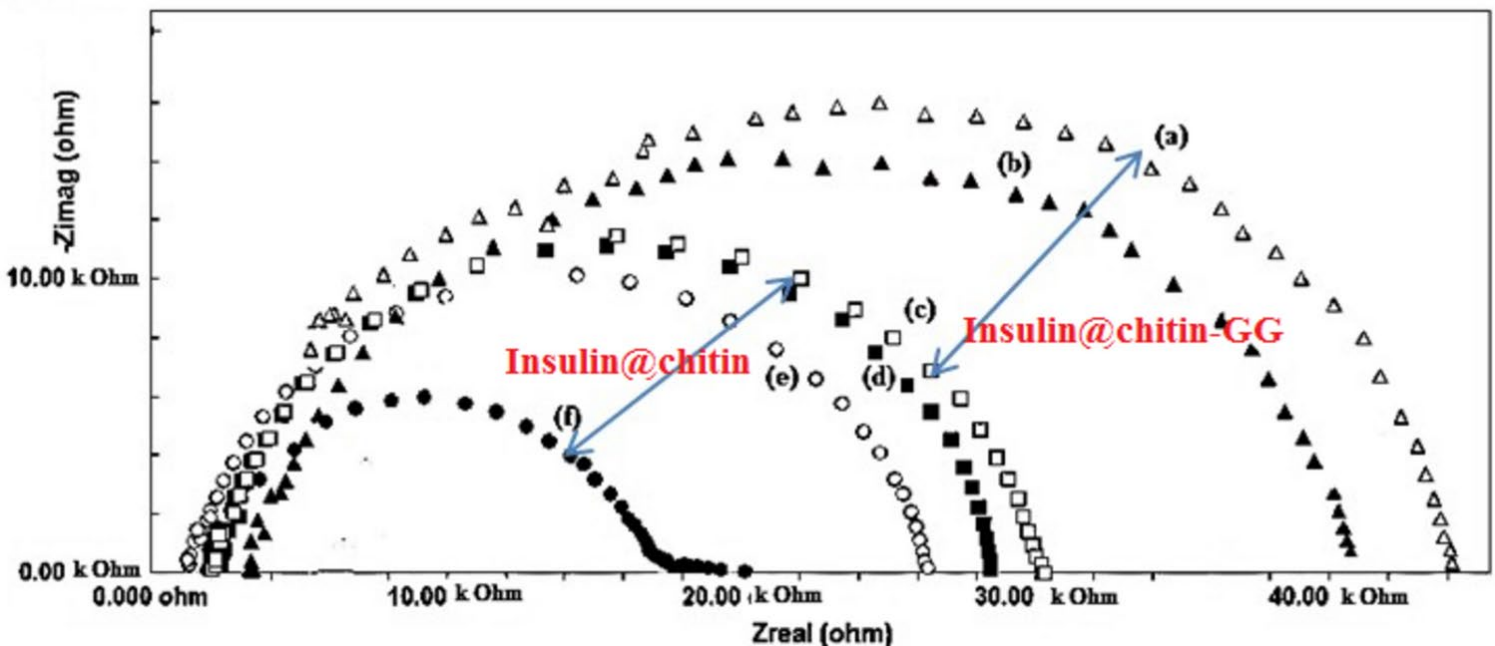
Impedance plots: insulin NCs at pH 1.2, 7.4 and 6.8 respectively.

The linear fitting of the release data to the pseudo 2° order model indicated insulin@chitin-g-GG heterogeneous solid surface has different energetic active sites^34^. Insulin molecule is release from two adsorption sites. The values of the rate constant k_2_ followed the order: pH 6.8 > 7.4 > 1.2 (confirmed by impedance plots, Fig. 10

Smaller charge transfer resistance (R_ct_ semicircle diameter) for insulin @chitin-GG at all pH indicated that low released [insulin] due to tight linkage to chitin. Few insulin molecules liberated from insulin@chitin adsorbed on WE surface by heteroatoms of FGs. DDS polarized by applied AC current, liberated insulin that adsorbed on surface of WE and increased R_ct_^29^. Release retarded at pH 1.2 and enhanced at pH 7.4. For insulin @chitin-GG, slow release at low pH indicating insulin protection against aggressive HCl in GIT: pH 1–3 (extend transit time till passage to small intestine for absorption in blood. Chitin is muco adhesive polymer carrier retard: insulin degradation and loss in gastric acid and intestine fluid. High release rate at colon pH 6.8 due to weakness insulin-NPs connections causing release without degradation. Absorption enhancers in mosaic layer and enterocytes connected by tight junction improved release. Insulin release is pH dependent. Similar pH response, such as insulin retention within polymer matrix at acidic pH and significant release at higher pH insulin alginate microspheres^12,26^.

AC current polarized (by amphoteric Zwitter ionic NH_2_ of insulin) and changed dielectric properties in IDDs^6,34^. Capacitive impedance plots confirmed: macroscopic dielectric properties, charge separation and phase transition^35^. Muco adhesive GG enhanced controlled sustained insulin release by hydrophilicity, charge density and pH response^34^. Insulin release via swelling by aqueous buffer solution. Insulin relaxation gives one time constant. EIS agreed with UV–vis. spectra: chitin carrier difficultly release insulin. Better insulin release from chitin-GG is due to swelling and porosity^21,22^, Table 6.

**Table 6:** Porosity and swelling percentage.

IDDs	Chitin	GG	Insulin @chitin	Insulin @chitin-g-GG
Porosity	503 ± 20	723 ± 37	63.37 ± 20	47.37 ± 20
Swelling%	3100	2900	980	650

Chitin had the largest swelling percentage. GG extensively form intramolecular H.B. (4 H.B. per monomer) forming 3D network structure. High rheology limited water absorption. Although swelling insulin @chitin exceeded insulin@chitin-g-GG, however strong linkage between semi crystalline chitin and insulin retard insulin release^21^. High porosity GG was attributed strong intermolecular H.B. Low porosity insulin @chitin-g-GG although better release suggested high: cross linking and loading capacity. Insulin loaded by both penetration into pores and binding multiple FGs. However, chitin-g-GG copolymer is loaded by more insulin decreased porosity^22^. Swelling, increase interior accessible surface for absorption aqueous physiological body fluid. Insulin @chitin-g-GG has hydrogel character behave as low viscous liquid with rapid phase transition. Grafted chitin was loaded by more insulin than chitin due to multiple FGs^22^.The aa test (8 h immersion in phosphate buffer saline) confirmed more peptides and aa sequence in insulin@chitin-g-GG^21,22^, Table 7.

**Table 7:** Peptides and aa sequence for IDDS.

	Insulin@chitin			Insulin@chitin-g-GG		
C _mg/mL_	1	3	8	1	3	8
Peptide	0.39	0.43	0.50	0.61	1.46	2.27
aa	0.05	0.51	0.9	0.071	0.69	1.04

Insulin (biological polypeptide protein; Mw. 5.800 k Da) degradation by ninhydrin (breaking peptide bonds between aa) giving aa (different NH_2_, COOH) position. Insulin exists in solution as monomers, dimers, tetramers and hexamers by ions-, and solvent interaction. Monomers are nonpolar hydrophobic, polar, neutral, basic, and acidic^21,22^. Insulin has primary protein structure (aa chains) has neither α-helix pleated sheet nor 3D conformation without association.

**Fig. 11:**
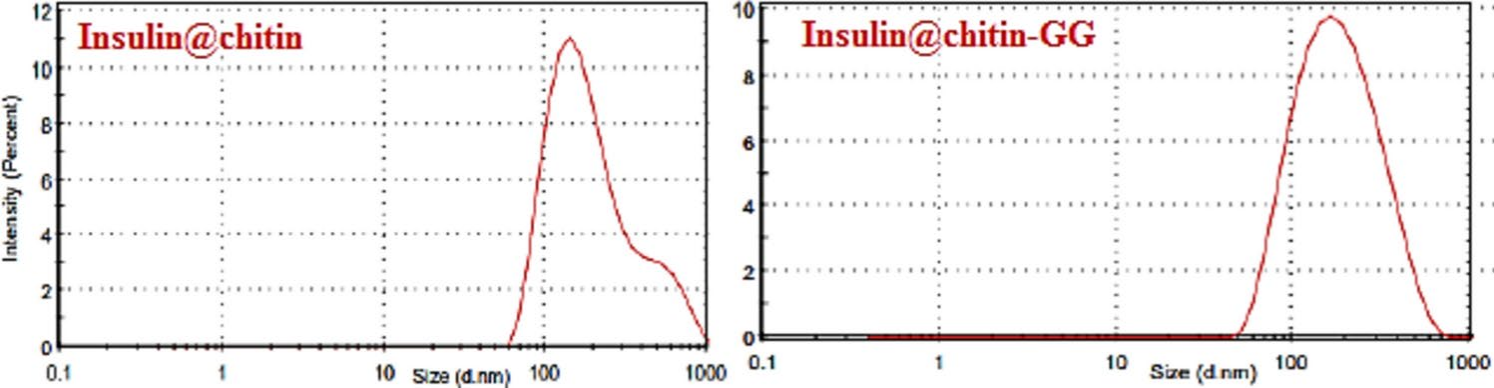
Particle size distributions.

Figure 11 showed that IDDS have the same particle size distribution between nano-, micro scale (70–1000 nm) up to 1 μm confirmed successful insulin loading on carrier.

P.S.D. is slightly modified at µm region for insulin@chtin-g-GG due to increased surface area by additional FGs^25^.

**Fig. 12:**
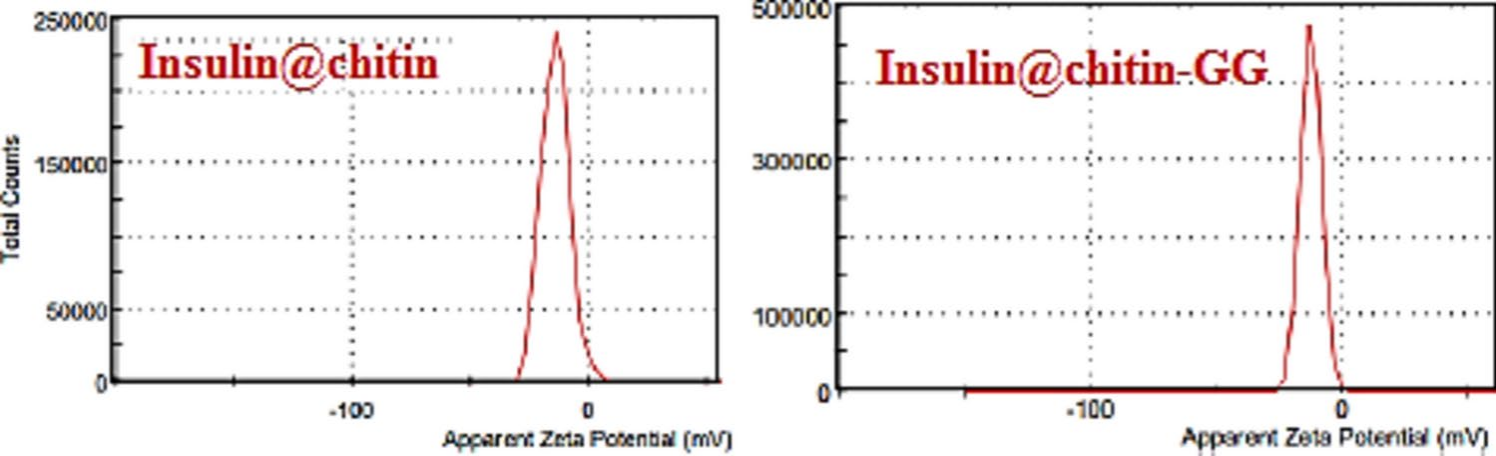
Zeta potentials insulin NCs.

Negative zeta potential originated from electrical double layer (Fig. 12) confirmed stability against: coagulation, storage temperatures, and mechanical shear force^25^.

Chitin-g-GG is ether copolymer hydrophilic arranged in block, random or alternating configuration throughout polymeric chains network, or interpenetrating polymer blends^36^. Insulin chains firmely linked to chitin NPs. Carbonyl acetamide group NHCOCH_3_ in chitin broken in alkaline reaction media, binding NH_2_-insulin. In insulin@chitin-g-GG, acloholic CH_2_OH of chitin form intermolecular H.B. with GG (intra-molecular) H.B^6^.

Insulin was chemical bonded and immobilized on the carrier polymers The chemical bonding between insulin and the carrier is more likely occurs via the functional groups and aided by chemical cross-linking, intermolecular hydrogen bonding, in addition to the electrostatic, hydrophobic and Van der Waals forces^34^. The electrostatic interactions between the acidic amino acids in insulin protein and acetyl groups of CT^35^.

## Conclusion

Chitin or chitin-GG are inert biodegradable permeable insulin carriers. Insulin loaded on chitin or its high functionality copolymer. Insulin-chains aa sequence bounded chitin decreased chains rigidity. Insulin@chitin has depth pores. Smoother insulin@chitin-g-GG had multiple FGs and little pores. Polymer chains chitin and GG cross linked via intermolecular H.B and Van der Waals interaction. Insulin@chitin semicrystalline with deep tunable pores. Chitin NH-, OH strongly binding insulin (O atom of COOH). Insulin chitin is more thermally stable. Insulin@chitin-g-GG showed better sustained insulin release. Chitin-g-GG is heterogeneous surface and each insulin molecule occupied 2.0 active sites. Targeting insulin-release at physiological temperature followed trend: pH 6.8 > pH 7.4 > pH 1.2 (lowest release rate at pH 1.2 indicated protection against acidic- or enzymatic hydrolysis in GIT, target sustained absorption in blood to regulate BGL. Physicochemical characteristics of insulin@chitin-g-GG and insulin@chitin porosity, swelling ratio, peptide &aa sequence, zeta potential, particle size distribution and release kinetics confirmed: Insulin loaded on chitin by chemisorbed monolayer and on chitin-g-GG by chemi-, and phyi-sorption. Electrostatic bonding to multiple FGs of copolymers facilitated insulin release. Chitin was electron acceptor Lewis acid. Chitin-g-GG was Lewis and Bronsted acid. Chitin had high insulin loading capacity. However, strong irreversible chemical bonding retard release.

## Supplementary Information

Supplementary Information 1.

Supplementary Information 2.

## Data Availability

All data generated or analysed during this study are included in this published article.
