# Supplementary material for: Formulation of new drug delivery systems for insulin from natural bioactive biocompatible polymers
Source: Sci Rep. 2025 Jan 31;15:3941. doi: 10.1038/s41598-025-86938-4 (PMC11785760; doi:10.1038/s41598-025-86938-4)
Supplement: Supplementary file 1 — Supplementary Information 1. [file 41598_2025_86938_MOESM1_ESM.docx]

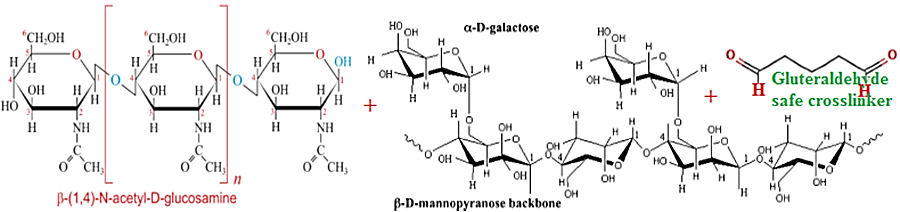


Fig.SI.1:Chemical structure of insulin carrier


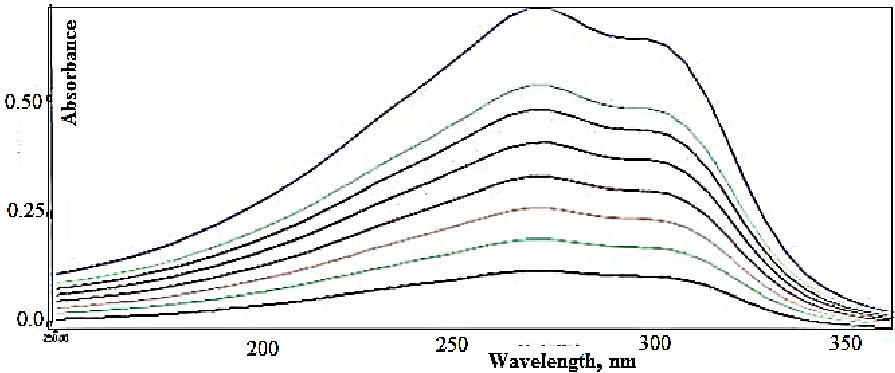


Fig. SI.2: Full curves UV-Vis. absorbance spectra

**
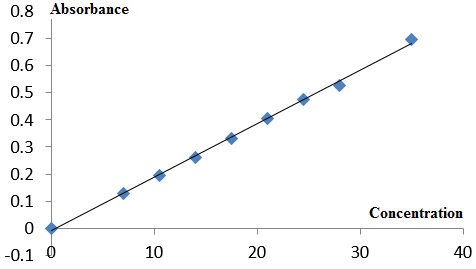
**

Fig. SI.3: Calibration curve of insulin


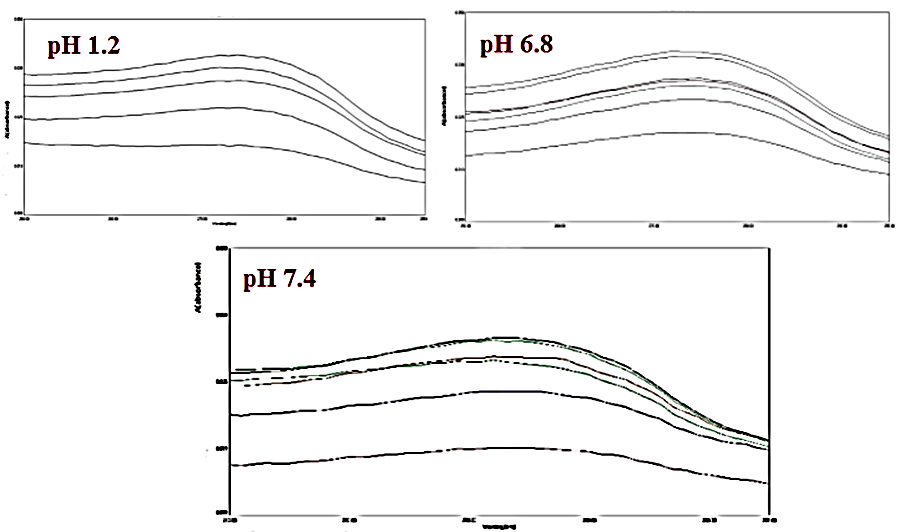


Fig. SI.4: Absorbance curves of insulin release at different pH 1.2, 6.8, 7.4


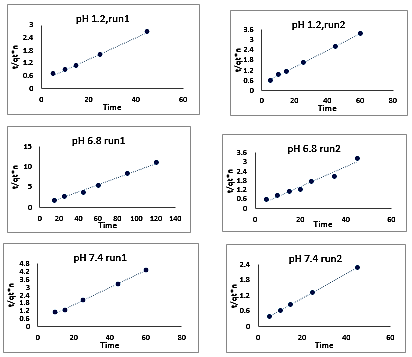


Fig.SI.5: Linear fitting of release insulin concentration to pseudo 2^o^ kinetic model

Table SI.1.Assigned functional groups from IR spectra

| $\bar{\upsilon},$cm^-1^ | Insulin @chitin | $\bar{\upsilon},$cm^-1^ | Insulin @chitin-guar gum |
| --- | --- | --- | --- |
| 3443.28 (Broad) | NH symmetric stretching, υ(OH), NH_2_ insulin | 3440.64 | NH symmetric stretching, υ(OH) GG and chitin, NH_2_ insulin |
| 2956.99 | υ_as_(CH_2_) | 3266.35 | sym stretching υ(OH) guar gum |
| 2892.45 | Peptide bond insulin | 3111.94 | υ(OH) guar gum (asym stretching) |
| 2096.35 | Acetyl group stretching | 2959.60 | Acetyl group stretching (chitin) |
| 1645.25 | asymmetric stretched C = O | 2923.49 | Peptide bond insulin |
| 1461.06 | υ(C-N) | 2888.67 | NH asymmetric stretching |
| 1413.80 | δ(CH_2_) | 2131.92 | υ(CH) |
| 1110.70 | CH_2_ rocking | 1659.98 | NH_2_ insulin |
| 1044.83 | CCN asymmetric stretching | 1629.48 | Peptide bond |
| 991.78 | CCN asymmetric stretching | 1559.42 | CH_2_OH |
| 920.11 | S-S insulin stretching | 1426.73 | Asymmetric stretched COO^-^ group |
| 677.81 | C = O bending, chitin; COO^-^ bending, insulin | 1378.37 | CH_2_ wagging |
| 1317.9287 | Waging of alcoholic OH, GG. | 1073.240 | (O….H), (O…. N) hydrogen bond chitin grafted GG |
| 1260.13 |  | 1027.904 |  |
| 1205.43 |  | 951.4857 |  |
| 1156.46 |  | 897.2575 |  |
| 1116.5159 | CH_2_ rocking | 700.7110 | COO- bending |
| 750.6411 | COO^-^ wagging |  |  |

Table SI.2: Values R^2^ for linear fitting release data to kinetic models

| Ritger-Peppas | Higuchi | Elovich | Pseudo 2^o^ | Pseudo 1^o^ | | 0^o^ | | Run | pH |
| --- | --- | --- | --- | --- | --- | --- | --- | --- | --- |
| 0.897 | 0.8452 | 0.943 | 0.9965 | 0.8845 | 0.7259 | | 1 | | 1.2 |
| 0.9609 | 0.9261 | 0.9857 | 0.9994 | 0.974 | 0.8338 | | 2 | |  |
| 0.6197 | 0.4794 | 0.5906 | 0.9959 | 0.3611 | 0.3691 | | 1 | | 6.8 |
| 0.6915 | 0.5293 | 0.6574 | 0.9618 | 0.3821 | 0.3977 | | 2 | |  |
| 0.8368 | 0.8098 | 0.8645 | 0.9975 | 0.7549 | 0.7466 | | 1 | | 7.4 |
| 0.9432 | 0.8815 | 0.9652 | 0.9998 | 0.7889 | 0.7721 | | 2 | |  |

Table SI.3: Parameters of regression analysis of released insulin to 2^o^ model

| pH 7.4 | | | pH 6.8 | | | pH 1.2 | | |
| --- | --- | --- | --- | --- | --- | --- | --- | --- |
| S.E. | R2 | Regression equation | S.E. | R^2^ | Regression equation | S.E. | R^2^ | Regression equation |
| 0.46 | 1.00 | y = 0.066x+0.325 | 0.32 | 1.00 | y = 0.088x+0.256 | 0.22 | 1.0 | Run1: y = 0.051x+ 0.36 |
| 0.58 | 1.00 | y = 0.048x+0.140 | 0.22 | 0.96 | y = 0.063x+0.138 | 0.25 | 1.0 | Run2:y = .050x+0.374 |
