# Supplementary material for: Formulation of new drug delivery systems for insulin from natural bioactive biocompatible polymers
Source: Sci Rep. 2025 Jan 31;15:3941. doi: 10.1038/s41598-025-86938-4 (PMC11785760; doi:10.1038/s41598-025-86938-4)
Supplement: Supplementary file 2 — Supplementary Information 2. [file 41598_2025_86938_MOESM2_ESM.docx]

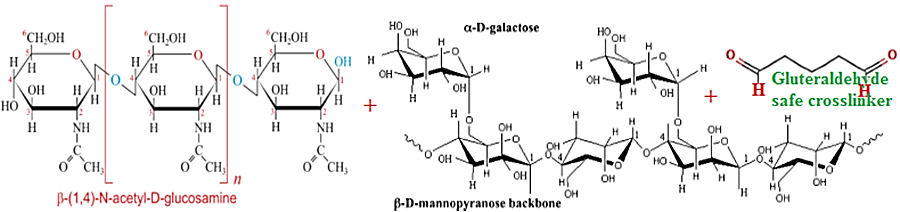


Fig.SI.1:Chemical structure of insulin carrier

Table SI.1.Assigned functional groups from IR spectra

| $\bar{\upsilon},$cm^-1^ | Insulin @chitin | $\bar{\upsilon},$cm^-1^ | Insulin @chitin-guar gum |
| --- | --- | --- | --- |
| 3443.28 (Broad) | NH symmetric stretching, υ(OH), NH_2_ insulin | 3440.64 | NH symmetric stretching, υ(OH) GG and chitin, NH_2_ insulin |
| 2956.99 | υ_as_(CH_2_) | 3266.35 | sym stretching υ(OH) guar gum |
| 2892.45 | Peptide bond insulin | 3111.94 | υ(OH) guar gum (asym stretching) |
| 2096.35 | Acetyl group stretching | 2959.60 | Acetyl group stretching (chitin) |
| 1645.25 | asymmetric stretched C = O | 2923.49 | Peptide bond insulin |
| 1461.06 | υ(C-N) | 2888.67 | NH asymmetric stretching |
| 1413.80 | δ(CH_2_) | 2131.92 | υ(CH) |
| 1110.70 | CH_2_ rocking | 1659.98 | NH_2_ insulin |
| 1044.83 | CCN asymmetric stretching | 1629.48 | Peptide bond |
| 991.78 | CCN asymmetric stretching | 1559.42 | CH_2_OH |
| 920.11 | S-S insulin stretching | 1426.73 | Asymmetric stretched COO^-^ group |
| 677.81 | C = O bending, chitin; COO^-^ bending, insulin | 1378.37 | CH_2_ wagging |
| 1317.9287 | Waging of alcoholic OH, GG. | 1073.240 | (O….H), (O…. N) hydrogen bond chitin grafted GG |
| 1260.13 |  | 1027.904 |  |
| 1205.43 |  | 951.4857 |  |
| 1156.46 |  | 897.2575 |  |
| 1116.5159 | CH_2_ rocking | 700.7110 | COO- bending |
| 750.6411 | COO^-^ wagging |  |  |
